# Treatment with *Commelina communis* Extract Exerts Anti-inflammatory Effects in Murine Macrophages via Modulation of the Nuclear Factor-*κ*B Pathway

**DOI:** 10.1155/2022/2028514

**Published:** 2022-02-24

**Authors:** Ji-Hee Kim, Bog-Im Park, Yong-Ouk You

**Affiliations:** ^1^Department of Convergence Technology for Food Industry, School of Food, Wonkwang University, Iksan, Jeonbuk, Republic of Korea; ^2^Department of Food and Nutrition, School of Food, Kunsan National University, Kunsan, Jeonbuk, Republic of Korea; ^3^Department of Oral Biochemistry, School of Dentistry, Wonkwang University, Iksan, Jeonbuk, Republic of Korea

## Abstract

The incidence of severe inflammatory diseases caused by chronic inflammation has increased owing to unprecedented changes brought about by industrialization. In this study, we aimed to assess the effect of treatment of lipopolysaccharide- (LPS-) induced murine macrophages with *Commelina communis* Linne extract (CCE) on synthesis of nitric oxide (NO), hypersecretion of proinflammatory cytokines, intranuclear transition of the p65 subunit of nuclear factor- (NF-) *κ*B, and degradation of the NF-*κ*B inhibitor I*κ*B*α*. Notably, CCE treatment did not affect cell viability even at a final concentration of 1.5 mg/mL. At a high concentration of CCE, the LPS-induced high levels of NO, tumor necrosis factor-*α*, interleukin- (IL-) 1*β*, and IL-6 were decreased via downregulation of inducible NO synthase and proinflammatory cytokine mRNA expression. Furthermore, phosphorylation of I*κ*B*α* was significantly decreased upon CCE treatment, and the intranuclear transition of NF-*κ*B p65 triggered by LPS was inhibited at a high concentration of CCE. Polyphenols and flavonoids, secondary metabolites in CCE that regulate the NF-*κ*B pathway, may be responsible for its anti-inflammatory activity. We suggest that CCE has anti-inflammatory effects related to suppression of the NF-*κ*B pathway and can be used to treat chronic inflammation.

## 1. Introduction

Inflammation is an innate defense response in living organisms. It protects the human body against pathogenic infections and provides adaptive immunity against specific pathogens. However, excessive inflammatory responses lead to serious tissue damage and various human diseases [1]. In recent times, humans are experiencing unprecedented environmental changes involving exposure to unhealthy diet, air pollution, fabrics made from synthetic fibers, and hygiene-related products that are harsh on the skin as well as ease of access to antibiotics, general pharmaceuticals, and quasi-drugs [2]. These changes in environmental factors may be unregulated and lead to induction of inflammatory processes that can in turn lead to homeostatic imbalance and pathologies such as chronic colitis [3], rheumatic diseases and arterial stiffness [4], fatty liver diseases [5], cancer [6], obesity and asthma [7], and sepsis [8]. The increase in human life expectancy in industrialized countries has resulted in a decrease in mortality. However, it has also increased the proportion of population that experiences the negative effects of chronic inflammatory diseases [2].

Macrophages play a key role in the initiation and maintenance of inflammation. They are stimulated upon exposure to cytokines (e.g., tumor necrosis factor-*α* (TNF-*α*)) and bacterial lipopolysaccharide (LPS) and invade the damaged tissue in large numbers. Here, the macrophages respond to various signals via their Toll-like receptors, which leads to their increased production of the transcription factor nuclear factor- (NF-) *κ*B, cytokines (e.g., TNF-*α*, interleukin- (IL-) 1*β*, and IL-6), and inducible nitric oxide synthase (iNOS), which in turn participate in regulation of the inflammatory response. These unstable metabolites adversely affect the integrity of the cell surface, and their overproduction leads to tissue damage and fibrosis [9, 10].

Therefore, it is important to develop effective and stable therapeutic strategies against chronic inflammation to alleviate pain in patients with inflammation-related diseases. Existing antiphlogistic drugs are commonly classified as steroidal (corticosteroids) and nonsteroidal (aspirin and ibuprofen) [11]. Although corticosteroids exhibit anti-inflammatory effects, they are associated with an increased incidence of acute coronary syndrome [12]. Nonsteroidal anti-inflammatory drugs alleviate pain and inflammation by blocking the enzyme cyclo-oxygenase; however, they also cause gastrointestinal bleeding, perforation, heart failure, and hyperkalemia. Administration of these drugs accounts for up to 30% of ulcer-related complications in patients [13]. Aspirin, too, has the potential to cause hepatotoxicity and anemia [14]. Considering the side effects of these preexisting drugs, there is currently a growing interest in the use of natural substances with anti-inflammatory properties as therapeutics [15].

The dayflower *Commelina communis* Linne belongs to the *Commelinaceae* family and is an annual monocotyledonous plant distributed throughout East Asia [16]. *C. communis* L. was widely used as an herbal medicine for sore throat, acute enteritis, obesity, and diabetes as well as a diuretic in medieval Korea [17]. The plant has also been traditionally used in Japan as a remedy for fever and diuresis [18]. Previous studies on *C. communis* L. have reported its antihyperglycemic activity [17], antioxidant activity [18], antiadipogenic effect [19], and anti-influenza virus activity [20]. *C. communis* L. may exert anti-inflammatory effects by obstructing the NF-*κ*B signaling pathway; however, the underlying mechanisms have not been reported. In this study, the effect of *C. communis* L. extract (CCE), a natural substance with low risk of side effects on the NF-*κ*B pathway, and its subsequent impact on anti-inflammatory response were investigated.

## 2. Materials and Methods

### 2.1. Preparation of CCE

**Figure 1:**
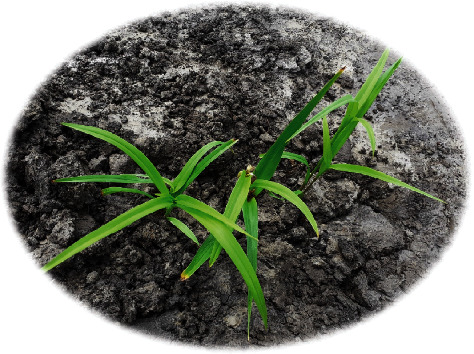
*Commelina communis* grown in a yard.

*Commelina communis* L. was purchased from a local market in Gurye, Jeonnam Province, Korea (Figure 1), and was dried. The dried *C. communis* L. was cut, 300 g of this sample was mixed with 3 L of 70% ethyl alcohol, and the mixture was subjected to cold extraction at 4°C. The filtered extract was subjected to evaporation for a week. A portion of the extract was used for analysis of secondary metabolites of CCE, and the rest of the extract was mixed with dimethyl sulfoxide (DMSO; Sigma-Aldrich Co., St. Louis, MO, USA) and stored at –20°C. The resultant product was used for subsequent experiments.

### 2.2. Analyses of CCE Components

#### 2.2.1. Total Polyphenol Content

Total polyphenol content was determined using the method reported by Blainski et al. [21], with a few modifications. Distilled water (150 *μ*L per well) was added to a 96-well plate (Thermo Fisher Inc., Waltham, MA, USA). Tannic acid (Sigma-Aldrich Co., Saint Louis, MO, USA), used as a polyphenol reference material, was diluted to 10–100 *μ*g/mL, and 20 *μ*L of each concentration was added to each well. Aliquots of 20 *μ*L CCE (1 mg/mL) were also added to each well. Thereafter, 10 *μ*L of 2 N Folin-Ciocalteu reagent (Sigma-Aldrich Co., Saint Louis, MO, USA) and 20 *μ*L of 35% sodium carbonate (Sigma-Aldrich Co., Saint Louis, MO, USA) solution were added. After incubating the plate for 1 h in the dark, the absorbance was measured at 760 nm wavelength using a microplate reader (Bio-Rad Laboratories, Inc., Irvine, CA, USA).

#### 2.2.2. Total Flavonoid Content

Total flavonoid content was estimated using the method reported by Chandra et al. [22]. CCE was diluted to 1 mg/mL with 80% ethanol, and 100 *μ*L of this solution was mixed with 100 *μ*L of 2% aluminum (III) chloride hexahydrate (Sigma-Aldrich Co., Saint Louis, MO, USA). Quercetin (Sigma-Aldrich Co., Saint Louis, MO, USA) was diluted in 80% ethanol from 2.5 *μ*g/mL to 100 *μ*g/mL. After mixing the diluted standard solution with 100 *μ*L of 2% aluminum (III) chloride hexahydrate, the mixture was incubated at room temperature for 10 min and then analyzed at 430 nm wavelength using a microplate reader.

#### 2.2.3. Total Anthocyanin Content

Total anthocyanin content was estimated using the method reported by Lee et al. [23], with a few modifications. KCl buffer (180 *μ*L of 0.025 M; pH 1.0 with HCl) was dispensed into each well of a 96-well plate. Then, cyanidin-3-glucoside chloride (Sigma-Aldrich Co., Saint Louis, MO, USA) was diluted to various concentrations (10–100 *μ*g/mL) with distilled water. The diluted extract (1 mg/mL) or standard solution (20 *μ*L) was added to the wells, and the plate was incubated at room temperature for 20 min. Absorbance was measured at 535 nm wavelength, and anthocyanins were quantified using the standard curve for cyanidin-3-glucoside chloride.

### 2.3. Cell Culture

RAW 264.7 macrophage cells purchased from the Korean Collection for Type Cultures (KCTC, Daejeon, Korea) were cultured in Roswell Park Memorial Institute (RPMI) 1640 medium supplemented with 2.05 mM l-glutamine, 10% heat-inactivated fetal bovine serum (Gibco, Thermo Fisher Scientific, Inc., Logan, UT, USA), and 1% antibiotic-antimycotic solution (Life technologies Co., Logan, UT, USA) at 37°C under optimal humidity and 5% carbon dioxide. The cells were subcultured every 2–3 days.

### 2.4. Analysis of Cell Viability

RAW 264.7 cells were cultured in a 24-well plate (Thermo Fisher Inc., Waltham, MA, USA) at 3 × 10^5^ cells/well for 24 h. The cells were treated with various concentrations of CCE (0.5–2 mg/mL) for 4 h. Diluted LPS solution (10 *μ*g/mL), at a final concentration of 100 ng/mL, was dispensed into each well. After incubating the plate at 37°C in a humidified atmosphere of 5% CO_2_ for 24 h, 3-(4,5-dimethyl-2-thiazolyl)-2,5-diphenyltetrazolium bromide solution (MTT; Duchefa biochemistry, Haarlem, Noord-Holland, Netherlands; final concentration 0.5 mg/mL) was added to each well, and the plate was incubated again for 4 h. Then, the medium and MTT solution were removed, and the formazan formed by macrophage cells was dissolved in DMSO and transferred into a 96-well plate for measuring the optical density at 540 nm wavelength. Cell viability (%) and CCE cytotoxicity were calculated relative to those in the control group that was not treated with the extract.

### 2.5. Quantification of NO

Cells were seeded into 24-well culture plates at 3 × 10^5^ cells/well and cultured at 37°C in 5% CO_2_ for 24 h. They were then treated with CCE (0.5–1.5 mg/mL) for 4 h. After a subsequent treatment with LPS (100 ng/mL) for 24 h, the supernatants were collected, and 100 *μ*L of each supernatant was added to 96-well plates. Subsequently, equal volume of Griess reagent (Promega, Madison, WI, USA) was added to the wells. The absorbance was measured at 540 nm wavelength. The NO yield was estimated using an NaNO_2_ calibration curve.

### 2.6. Quantification of Cytokines

Cell culture supernatant was obtained as described in Section 2.5 and assayed for TNF-*α*, IL-1*β*, and IL-6 using enzyme-linked immunosorbent assay (ELISA) kits (Becton, Dickinson and Company, San Diego, CA, USA) following the manufacturer's instructions. Briefly, a 96-well plate was precoated with the capture antibody at 4°C overnight, then washed with wash buffer followed by the addition of blocking buffer for 1 h. After rinsing the wells, 100 *μ*L of the culture supernatant or standard solution was added into each well, and the plate was incubated at room temperature for 2 h. After washing the plate, detection antibody was added, and the plate was once again incubated for 1 h; the wells were then washed to remove excess detection antibody. Subsequently, streptavidin–horseradish peroxidase- (HRP-) conjugated antibody was added, and the plate was incubated for 30 min, followed by rinsing. Then, 100 *μ*L of tetramethylbenzidine solution (Becton, Dickinson and Company, San Diego, CA, USA) was added into each well, and the plate was again incubated at room temperature in the dark for 30 min. Subsequently, 50 *μ*L of 1 M H_3_PO_4_ solution was added. Following completion of the reaction, the absorbance was measured at 450 nm wavelength. Cytokine production was determined using the standard curves of the respective standard reagents in the ELISA kits.

### 2.7. Real-Time Polymerase Chain Reaction (PCR)

RAW 264.7 cells were cultured in 56.7 cm^2^ cell culture plates (Thermo Fisher Inc., Waltham, MA, USA) as described in Section 2.5 and harvested by centrifuging at 1,000 rpm and 4°C for 5 min (Hanil Scientific Inc., Incheon, Korea). RNA was extracted and purified following the method reported by Lee et al. [24]. The cell pellets were treated with TRIzol^Ⓡ^ reagent (Life Technologies, Carlsbad, CA, USA) to obtain total RNA. Chloroform (0.2 mL) was added, and the plate was incubated for 10 min, and the transparent layer from the centrifuged sample was mixed with the same volume of isopropanol, and the mixture was incubated at 4°C for 10 min. After centrifugation, the white precipitate was washed with 70% ethanol. A moderate amount of diethylpyrocarbonate-treated water (USB Co., Cleveland, OH, USA) was added to the RNA. Purified RNA was quantified at 260 nm wavelength using a spectrophotometer (Shimadzu Co., Kyoto, Japan), and cDNA was synthesized using RevertAid™ First Strand cDNA Synthesis Kit (Thermo Scientific, Berlin, Germany) following the manufacturer's instructions. Subsequently, 1 *μ*L of cDNA and 0.5 *μ*M of primers were used to prepare 20 *μ*L of the PCR mixture using VeriQuest™ SYBR® Green qPCR Master Mix (Affymetrix, Inc., Cleveland, Ohio, USA). cDNA amplification was carried out using the StepOnePlus Real-time PCR system (Applied Biosystems, Foster City, CA, USA). The initial denaturation step was maintained at 95°C for 5 min, followed by 40 cycles of denaturation at 95°C for 15 s, annealing at 60°C for 1 min, and extension at 72°C for 30 s. The relative mRNA levels were determined using the *ΔΔ*Ct method and normalized to that of *β*-actin. The primer sequences are listed in Table 1.

### 2.8. Western Blotting

Cellular pellets of murine macrophages were obtained as described in Section 2.7. Nuclear proteins were isolated from RAW 264.7 cells using a nuclear protein extraction kit (Cayman Chemical Company, Ann Arbor, MI, USA) according to the manufacturer's protocol. Cytosolic proteins were extracted using PhosphoSafe Protein extraction reagent (Novagen, Inc., Gibbstown, NJ, USA) following the manufacturer's instructions. Proteins were quantitated using the bicinchoninic acid protein assay (Pierce Biotechnology, Waltham, MA, USA) at 562 nm wavelength. Twenty micrograms of each protein sample was separated using 10% sodium dodecyl sulfate-polyacrylamide gel electrophoresis. The separated proteins were transferred onto a polyvinylidene fluoride membrane (GE Healthcare, Little Chalfont, UK), blocked for 1 h in 5% skim milk, and then incubated with diluted primary antibodies (1 : 1,000; Cell Signaling, Danvers, MA, USA) overnight at 4°C on a shaker (DAIHAN scientific, Namyangju, South Korea). Subsequently, HRP-conjugated secondary antibodies (1 : 2,500; Cell Signaling, Danvers, MA, USA) diluted in 3% skim milk were added to the membrane, which was then incubated for 2 h. After washing the membrane, protein bands were visualized using an enhanced chemiluminescence kit (Advansta Inc., San Jose, CA, USA) and imaged using a chemiluminescence imaging system (Azure Biosystems, Inc., Sierra, CA, USA). Protein expression levels were quantified using the ImageJ analysis software (National Institutes of Health, Bethesda, MD, USA).

### 2.9. NF-*κ*B Transcription Factor Assay

Nuclear extracts were prepared as described in Section 2.8 and then assessed for the DNA-binding capability of NF-*κ*B p65 following the protocol described in the TransAM™ NF-*κ*B p65 kit (Active Motif, Inc., Carlsbad, CA, USA). Briefly, standard solutions and nuclear extracts were added to each well of the assay plate, which was then incubated for 1 h at 100 rpm on a rocker. After washing the wells with wash buffer, primary antibodies for NF-*κ*B subunits were added, and the plate was incubated for 1 h at room temperature followed by another wash step. Subsequently, HRP-conjugated secondary antibody was added to each well, and the plate was again incubated for 1 h and then rinsed. Then, 100 *μ*L of developing solution was dispensed into each well, and the reaction terminated by adding 100 *μ*L of stop solution for approximately 5 min. The absorbance of the reaction solution was then measured at 450 nm wavelength. NF-*κ*B p65 translocation into the nucleus was calculated using the standard curve of the standard reagent in the NF-*κ*B p65 kit.

### 2.10. Statistical Analysis

All experiments were repeated three times. Statistical analysis was performed using Student's *t*-test and one-way analysis of variance (ANOVA) in Microsoft® Excel, and the results are expressed as the mean ± standard deviation. The criterion for statistical significance was set at *P* < 0.05.

## 3. Results

### 3.1. Phytochemical Components of CCE

The total polyphenol, flavonoid, and anthocyanin contents of CCE are presented in Table 2. The total polyphenol content was as high as 19.1 ± 0.4 mg tannic acid equivalent (TAE)/g, total flavonoid content was 6.0 ± 0.0 mg quercetin equivalent (QE)/g, and total anthocyanin was not detected.

### 3.2. CCE Cytotoxicity and Effect on RAW 264.7 Macrophage Viability

**Figure 2:**
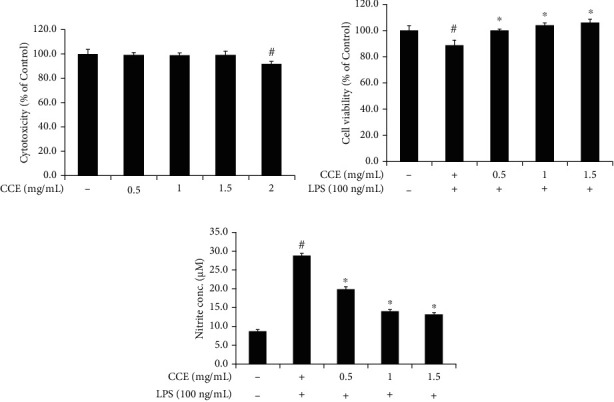
*Commelina communis* extract (CCE) treatment restricts nitric oxide (NO) production in lipopolysaccharide- (LPS-) exposed RAW 264.7 cells. (a) Cytotoxicity, (b) viability, and (c) NO levels of murine macrophages. Murine macrophages were treated with 100 ng/mL LPS. The cytotoxicity and cellular viability were measured using MTT assay, and NO levels were evaluated using Griess reaction. Data were compared between groups using Student's *t*-test and one-way analysis of variance. The results are expressed as the mean ± standard deviation from three independent experiments. ^#^*P* < 0.05 compared with the control; ^∗^*P* < 0.05 compared with the LPS-exposed group.

The cytotoxicity of CCE at 0.5, 1, 1.5, and 2 mg/mL was 99.17 ± 1.80%, 99.01 ± 1.70%, 99.37 ± 2.77%, and 91.77 ± 2.05%, respectively, compared with that in the untreated control group (Figure 2(a)). The group treated with LPS for 24 h showed a significantly reduced cell survival rate of 88.68 ± 3.90% compared with the control group. The viability of the cells treated with CCE was enhanced in a dose-dependent manner compared with that in the LPS-treated group (Figure 2(b)).

### 3.3. Suppression of NO Synthesis in RAW 264.7 Cells Treated with CCE

**Figure 3:**
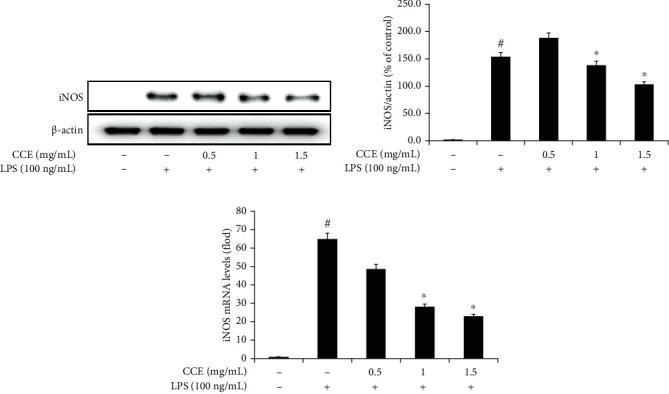
*Commelina communis* extract (CCE) treatment downregulates inducible nitric oxide synthase (iNOS) expression in lipopolysaccharide- (LPS-) treated RAW 264.7 cells. iNOS expression in cytosolic protein fraction was examined using western blotting (a) and quantitated upon comparison with *β*-actin expression (b). iNOS mRNA expression was investigated using real-time polymerase chain reaction (c). Data are presented as the mean ± standard deviation from three independent experiments. ^#^*P* < 0.05 compared with the control; ^∗^*P* < 0.05 compared with the LPS-exposed group.

NO production was considerably higher in the LPS-treated group than in the untreated group. Nitrite generation in the CCE-treated group decreased in a dose-dependent manner compared with that in the LPS-treated group (Figure 2(c)). NO synthesis is regulated by the iNOS expression in LPS-exposed macrophages [25]. In the LPS-treated group, the iNOS mRNA expression and cytosolic protein levels were significantly increased compared with those in the untreated group, which was reversed after treatment with various concentrations of CCE. A significant reduction in the iNOS expression compared with that in the LPS-stimulated group was observed following treatment with 1 mg/mL and 1.5 mg/mL of CCE (Figure 3).

### 3.4. Inhibition of Proinflammatory Cytokine Secretion in RAW 264.7 Macrophages Treated with CCE

**Figure 4:**
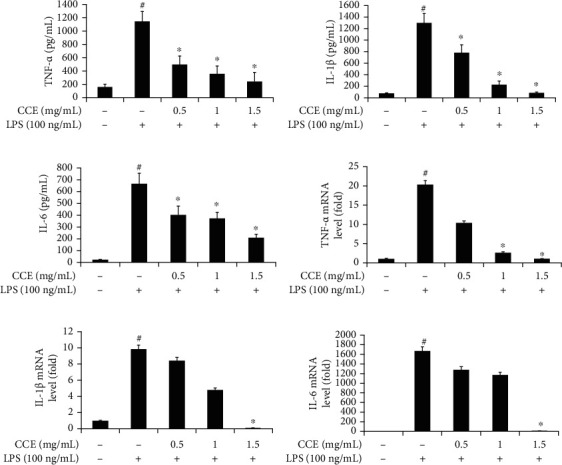
Effect of *Commelina communis* extract (CCE) treatment on lipopolysaccharide- (LPS-) induced production of cytokines such as tumor necrosis factor- (TNF-) *α*, interleukin- (IL-) 1*β*, and IL-6 in RAW 264.7 cells; protein expression (a–c) and mRNA expression (d–f). Cells were pretreated with the indicated concentrations of CCE (0.5, 1, and 1.5 mg/mL) for 4 h and then exposed to LPS for 24 h. Cell supernatants were analyzed using enzyme-linked immunosorbent assay and real-time polymerase chain reaction. The experimental results, in triplicate, are indicated as the mean ± standard deviation. ^#^*P* < 0.05 compared with the control; ^∗^*P* < 0.05 compared with the LPS-exposed group.

To examine whether CCE treatment affects cytokine secretion in LPS-exposed RAW 264.7 cells, the mRNA and protein expression levels of TNF-*α*, IL-1*β*, and IL-6 were determined using real-time PCR and ELISA, respectively. The protein and mRNA levels of TNF-*α*, IL-1*β*, and IL-6 were downregulated in a dose-dependent manner (Figure 4). In particular, the CCE-treated group showed a significant reduction at specific concentrations: 1 and 1.5 mg/mL of CCE for TNF-*α* and 1.5 mg/mL of CCE for IL-1*β* and IL-6.

### 3.5. Inhibition of Intranuclear Transition of NF-*κ*B p65 under CCE Treatment

The NF-*κ*B p65 subunit is part of the predominant pathway involved in the secretion of proinflammatory molecules [26]. TLR4 response to LPS stimulation causes an influx of the NF-*κ*B p65 subunit into the nucleus [27].

**Figure 5:**
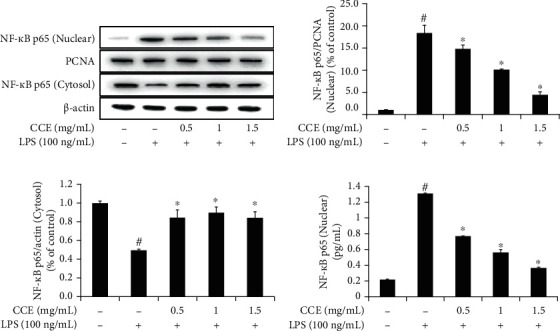
*Commelina communis* extract (CCE) treatment hinders activation of the p65 subunit of nuclear factor- (NF-) *κ*B in RAW 264.7 cells. Murine macrophages were treated with CCE for 4 h and then exposed to lipopolysaccharide (LPS) for 1 h. (a) NF-*κ*B p65 expression levels in the nuclear and cytosolic protein fractions were detected using western blotting and quantified using standard solutions of PCNA (b) and *β*-actin (c), respectively. (d) Nuclear NF-*κ*B was assessed using enzyme-linked immunosorbent assay. Data are expressed as the mean ± standard deviation from three independent experiments. ^#^*P* < 0.05 compared with the control; ^∗^*P* < 0.05 compared with the LPS-exposed group.

In the untreated group, the cytoplasmic content of NF-*κ*B p65 was high. Upon exposure to LPS, the nuclear content of the p65 protein subunit was markedly increased. When the protein content in the cytoplasm was increased following treatment with a high concentration of CCE, the translocation of the p65 subunit into the nucleus decreased (Figure 5).

### 3.6. Inhibition of I*κ*B*α* Phosphorylation under CCE Treatment

**Figure 6:**
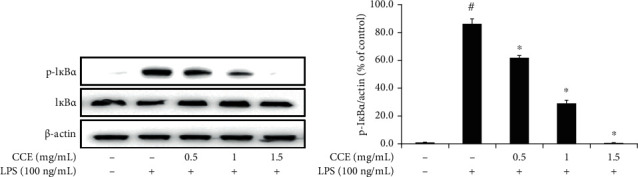
*Commelina communis* extract (CCE) treatment suppresses I*κ*B*α* phosphorylation. Murine macrophages were treated with CCE for 4 h and then exposed to lipopolysaccharide (LPS) for 1 h. The expression of both phospho- (p-) and total I*κ*B*α* in the cytosolic protein fraction was evaluated using western blotting (a) and quantitated using the relative expression of *β*-actin (b). Data are shown as the mean ± standard deviation from three independent experiments. ^#^*P* < 0.05 compared with the control; ^∗^*P* < 0.05 compared with the LPS-exposed group.

Transcriptional activation of NF-*κ*B occurs when the protein I*κ*B*α* is phosphorylated. The phosphorylation of I*κ*B*α* leads to dissociation of the NF-*κ*B complex, which is then translocated to the nucleus [28]. LPS treatment induced an increase in I*κ*B*α* phosphorylation, which was noticeably reduced upon treatment with increasing concentrations of CCE (*P* < 0.05) (Figure 6).

## 4. Discussion

Inflammatory diseases, such as asthma, rheumatoid arthritis, atherosclerosis, and diabetes, arise from excessive NO and cytokine production via the NF-*κ*B pathway [29–31]. RAW 264.7 macrophages treated with LPS have been commonly used as a cellular model of inflammation. Macrophages are activated by LPS resulting in the induction of proinflammatory cytokines and inflammatory responses via the NF-*κ*B signaling pathway [29, 30]. Murine macrophages respond to stimulation with LPS from *Escherichia coli* via their TLR4 [32]. TNF-*α* or LPS induces rapid decomposition and phosphorylation of I*κ*B*α*. NF-*κ*B binds to I*κ*B*α* to form a complex in the cytoplasm. I*κ*B*α* kinase, ubiquitin ligase, and other factors are involved in the regulation of I*κ*B*α* phosphorylation and degradation [33, 34]. The NF-*κ*B protein family is composed of RelA/p65 and p105/p50. RelA/p65 has a transactivation-dependent site and is responsible for the transcription activity of NF-*κ*B. I*κ*B*α* degradation causes p65 to be translocated into the nucleus thereby activating NF-*κ*B [33]. Subsequently, NF-*κ*B activation induces the transcription of many proinflammatory genes (those encoding iNOS, TNF-*α*, and IL-6). An increased amount of NO is produced from l-arginine by iNOS [35]. IL-1*β* chiefly activates the *IL-6* promoter through the NF-*κ*B pathway to induce IL-6 production [36].

Recently, a few studies investigated various natural substances for their anti-inflammatory effects and potential in the treatment of inflammatory conditions [37, 38]. In this study, we assessed whether a 70% ethyl alcohol extract of *C. communis* L. has an inhibitory effect on LPS-stimulated inflammation in RAW 264.7 macrophages.

MTT assay was performed to determine whether CCE cytotoxicity impairs the expression of proinflammatory factors of the NF-*κ*B pathway. Cytotoxicity analysis of CCE showed cell viability above 99% at concentrations ranging from 0.5 mg/mL to 1.5 mg/mL compared with that in the untreated control (Figure 2(a)). The mechanism of the anti-inflammatory effect of CCE via the NF-*κ*B pathway was investigated *in vitro*, and 1.5 mg/mL of CCE was determined to be the optimal concentration for treatment.

In the immune system, NO exhibits antimicrobial and antiviral effects. Moreover, cytokine or bacterial pathogen stimulation activates iNOS. NO is synthesized from l-arginine and is overproduced by the activation of NF-*κ*B. High concentrations of NO often cause cell and tissue damage by generating reactive radicals such as peroxynitrate and mediate inflammatory diseases [35, 39, 40]. In this study, CCE treatment decreased NO production as well as the mRNA and cytosolic protein levels of iNOS secreted by LPS-exposed macrophages in a dose-dependent manner (Figures 2(c) and 3), indicating significant differences between the results of the CCE- and LPS-treated groups at concentrations between 1 mg/mL and 1.5 mg/mL. This suggests that CCE, an anti-inflammatory agent, is capable of reducing iNOS and NO production and preventing inflammation-related tissue damage by regulating the iNOS gene expression at a concentration of 1 mg/mL or more (Figure 3).

TNF-*α* promotes the initiation of inflammation by activating T cells and macrophages and can lead to the development of atherosclerosis and glucose metabolic disorders [25, 35]. IL-1*β* and IL-6, cytokines that amplify acute inflammation, trigger autoimmune reactions that can evolve into a chronic inflammatory condition [41, 42]. In this study, we tested whether CCE treatment reduced the secretion of proinflammatory cytokines. The relative mRNA and protein levels of TNF-*α*, IL-1*β*, and IL-6 were downregulated in a dose-dependent manner (Figure 4). Overall, compared with those for the LPS-treated control in the cytokine assay and real-time PCR experiments, the mRNA levels of TNF-*α* were significantly decreased at CCE concentrations greater than 1 mg/mL. The relative mRNA levels of IL-1*β* and IL-6 were significantly affected by 1.5 mg/mL of CCE treatment (*P* < 0.05). Therefore, CCE, a preliminary anti-inflammatory agent, might be capable of blocking the secretion of proinflammatory cytokines.

NF-*κ*B induces expression of diverse proinflammatory genes in innate and adaptive immunity, and activated NF-*κ*B can mediate the pathogenesis of inflammatory diseases [30]. We analyzed the effects of CCE treatment on NF-*κ*B activation and I*κ*B*α* degradation to understand the potential anti-inflammatory efficacy of CCE through modulation of the NF-*κ*B pathway. LPS treatment increased the levels of NF-*κ*B p65 translocated into the nucleus and the phosphorylation of I*κ*B*α* (Figures 5 and 6). Furthermore, I*κ*B*α* phosphorylation and nuclear p65 subunit levels were inhibited by CCE treatment in a dose-dependent manner. These results suggested that CCE treatment inhibits the intranuclear transition of the NF-*κ*B p65 subunit by regulating the upstream events of I*κ*B*α* phosphorylation and decomposition. Anti-inflammatory substances in plants are secondary metabolites; they include phenolic compounds, tannins, flavonoids, alkaloids, sterols, terpenoids, and essential oils [43]. Polyphenols are known to reduce the levels of proinflammatory cytokines by inactivating TLR4 and affect the interaction of NF-*κ*B with DNA. Flavonoids inhibit the phosphorylation and decomposition of I*κ*B*α* and obstruct the translocation of NF-*κ*B p65 into the nucleus, thereby impeding the expression of iNOS [44]. We found that the total polyphenol content of CCE was the highest at 19.1 ± 0.4 mg TAE/g, and the total flavonoid content was 6.0 ± 0.0 mg QE/g. Therefore, CCE was determined to be rich in polyphenols and flavonoids. Taken together, these findings indicate that the anti-inflammatory activity of CCE in LPS-exposed RAW 264.7 macrophages can be attributed to its secondary metabolites, which serve to inhibit the NF-*κ*B pathway.

## 5. Conclusion

**Figure 7:**
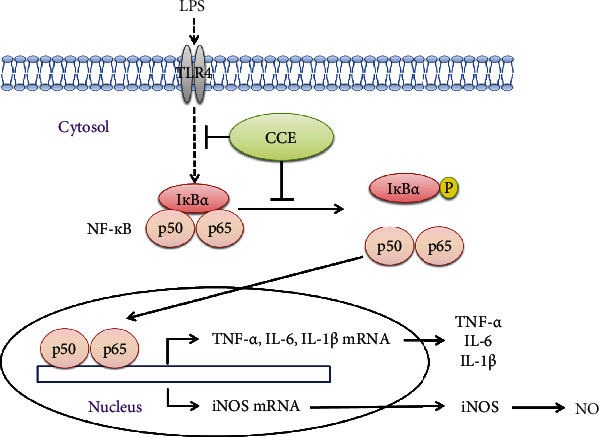
Mechanism of *Commelina communis* extract- (CCE-) induced suppression of lipopolysaccharide- (LPS-) mediated inflammatory reactions in RAW 264.7 cells.

CCE may be involved in suppressing inflammatory activity in LPS-induced murine macrophages by downregulating inflammatory mediators involved in the NF-*κ*B pathway (Figure 7).

The results of this study may provide the basis for development of natural therapies against inflammatory diseases caused by chronic inflammation, and CCE may serve as the leading anti-inflammatory candidate agent.

## Figures and Tables

**Table 1:** Primer sequences used for real-time polymerase chain reaction and conditions for detection of proinflammatory gene expression.

Genes	Sequence (5′-3′)	Size (bp)	Tm (°C)	Cycles
TNF-*α*	Forward: 5′-GGCAGGTCTACTTTGGAGTCATTGC-3′ Reverse: 5′-ACATTCGAGGCTCCAGTGAATTCGG-3′	305	65	40
IL-1*β*	Forward: 5′-GAAAGACGGCACACCCACCCT-3′ Reverse: 5′-GCTCTGCTTGTGAGGTGCTGATGTA-3′	167	65	40
IL-6	Forward: 5′-TCAGAATTGCCATTGCACA-3′ Reverse: 5′-GTCGGAGGCTTAATTACACATG-3′	74	55	45
iNOS	Forward: 5′-AAGTCAAATCCTACCAAAGTGA-3′ Reverse: 5′-CCATAATACTGGTTGATGAACT-3′	409	65	40
*β*-Actin	Forward: 5′-CATCACTATCGGCAATGAGC-3′ Reverse: 5′-GACAGCACTGTGTTGGCATA-3′	159	65	40

**Table 2:** Secondary metabolite contents of *Commelina communis* extract.

Phytochemicals	Contents
Total polyphenol (mg TAE^a^/g)	19.1 ± 0.4^d^
Total flavonoid (mg QE^b^/g)	6.0 ± 0.0
Total anthocyanin (mg C3G^c^/g)	N.D.^e^

^a^TAE: tannic acid equivalent. ^b^QE: quercetin equivalent. ^c^C3G: cyanidin-3-glucoside equivalent. ^d^The total value is expressed as the mean ± standard deviation (SD) from three independent experiments. ^e^Not detected.

## Data Availability

No data were used to support this study.
